# Supplementary material for: Unveiling the Bioactive Potential of the Invasive Jellyfish Phyllorhiza punctata Through Integrative Transcriptomic and Proteomic Analyses
Source: Biomolecules. 2025 Aug 4;15(8):1121. doi: 10.3390/biom15081121 (PMC12383608; doi:10.3390/biom15081121)
Supplement: Supplementary file 1 [file biomolecules-15-01121-s001.zip › Suplementary Material/FileS3/PEPFOLD4/XP_065056045_11954/clusters.html]

 PEP-FOLD clusters


Download archive of all models  here   

|  |  |  |
| --- | --- | --- |
| # | file | sOPEP |
| # cluster 1 - Size: 17 |  |  |
|  | XP\_065056045\_11954-model1 | -17.7175 |
|  | XP\_065056045\_11954-model1.1 | -17.7043 |
|  | XP\_065056045\_11954-model1.2 | -17.6214 |
|  | XP\_065056045\_11954-model1.3 | -17.5119 |
|  | XP\_065056045\_11954-model1.4 | -17.4199 |
|  | XP\_065056045\_11954-model1.5 | -17.4183 |
|  | XP\_065056045\_11954-model1.6 | -17.272 |
|  | XP\_065056045\_11954-model1.7 | -16.0825 |
|  | XP\_065056045\_11954-model1.8 | -15.83 |
|  | XP\_065056045\_11954-model1.9 | -15.8027 |
|  | XP\_065056045\_11954-model1.10 | -15.706 |
|  | XP\_065056045\_11954-model1.11 | -15.6829 |
|  | XP\_065056045\_11954-model1.12 | -15.5713 |
|  | XP\_065056045\_11954-model1.13 | -15.4494 |
|  | XP\_065056045\_11954-model1.14 | -15.2741 |
|  | XP\_065056045\_11954-model1.15 | -14.9795 |
|  | XP\_065056045\_11954-model1.16 | -13.5315 |
| # cluster 2 - Size: 13 |  |  |
|  | XP\_065056045\_11954-model2 | -17.1955 |
|  | XP\_065056045\_11954-model2.1 | -17.1377 |
|  | XP\_065056045\_11954-model2.2 | -16.512 |
|  | XP\_065056045\_11954-model2.3 | -16.4799 |
|  | XP\_065056045\_11954-model2.4 | -16.463 |
|  | XP\_065056045\_11954-model2.5 | -16.2415 |
|  | XP\_065056045\_11954-model2.6 | -16.148 |
|  | XP\_065056045\_11954-model2.7 | -16.1137 |
|  | XP\_065056045\_11954-model2.8 | -15.9413 |
|  | XP\_065056045\_11954-model2.9 | -15.9059 |
|  | XP\_065056045\_11954-model2.10 | -15.7306 |
|  | XP\_065056045\_11954-model2.11 | -15.722 |
|  | XP\_065056045\_11954-model2.12 | -15.5916 |
| # cluster 3 - Size: 2 |  |  |
|  | XP\_065056045\_11954-model3 | -16.7771 |
|  | XP\_065056045\_11954-model3.1 | -15.8526 |
| # cluster 4 - Size: 14 |  |  |
|  | XP\_065056045\_11954-model4 | -16.7165 |
|  | XP\_065056045\_11954-model4.1 | -16.7165 |
|  | XP\_065056045\_11954-model4.2 | -16.6685 |
|  | XP\_065056045\_11954-model4.3 | -16.5939 |
|  | XP\_065056045\_11954-model4.4 | -16.5719 |
|  | XP\_065056045\_11954-model4.5 | -16.3227 |
|  | XP\_065056045\_11954-model4.6 | -16.2719 |
|  | XP\_065056045\_11954-model4.7 | -16.166 |
|  | XP\_065056045\_11954-model4.8 | -15.9071 |
|  | XP\_065056045\_11954-model4.9 | -15.7369 |
|  | XP\_065056045\_11954-model4.10 | -15.4992 |
|  | XP\_065056045\_11954-model4.11 | -15.4793 |
|  | XP\_065056045\_11954-model4.12 | -15.4648 |
|  | XP\_065056045\_11954-model4.13 | -15.3845 |
| # cluster 5 - Size: 3 |  |  |
|  | XP\_065056045\_11954-model5 | -16.5185 |
|  | XP\_065056045\_11954-model5.1 | -16.4849 |
|  | XP\_065056045\_11954-model5.2 | -15.7243 |
| # cluster 6 - Size: 4 |  |  |
|  | XP\_065056045\_11954-model6 | -16.063 |
|  | XP\_065056045\_11954-model6.1 | -16.0277 |
|  | XP\_065056045\_11954-model6.2 | -15.9663 |
|  | XP\_065056045\_11954-model6.3 | -15.3097 |
| # cluster 7 - Size: 2 |  |  |
|  | XP\_065056045\_11954-model7 | -15.9072 |
|  | XP\_065056045\_11954-model7.1 | -13.6122 |
| # cluster 8 - Size: 12 |  |  |
|  | XP\_065056045\_11954-model8 | -15.4586 |
|  | XP\_065056045\_11954-model8.1 | -15.4144 |
|  | XP\_065056045\_11954-model8.2 | -15.3465 |
|  | XP\_065056045\_11954-model8.3 | -15.2732 |
|  | XP\_065056045\_11954-model8.4 | -15.2353 |
|  | XP\_065056045\_11954-model8.5 | -15.1387 |
|  | XP\_065056045\_11954-model8.6 | -14.9081 |
|  | XP\_065056045\_11954-model8.7 | -14.8556 |
|  | XP\_065056045\_11954-model8.8 | -14.6946 |
|  | XP\_065056045\_11954-model8.9 | -14.4676 |
|  | XP\_065056045\_11954-model8.10 | -14.3748 |
|  | XP\_065056045\_11954-model8.11 | -14.1705 |
| # cluster 9 - Size: 2 |  |  |
|  | XP\_065056045\_11954-model9 | -15.2246 |
|  | XP\_065056045\_11954-model9.1 | -15.0573 |
| # cluster 10 - Size: 1 |  |  |
|  | XP\_065056045\_11954-model10 | -14.3226 |
| # cluster 11 - Size: 2 |  |  |
|  | XP\_065056045\_11954-model11 | -13.2262 |
|  | XP\_065056045\_11954-model11.1 | -12.0091 |
| # cluster 12 - Size: 4 |  |  |
|  | XP\_065056045\_11954-model12 | -12.5491 |
|  | XP\_065056045\_11954-model12.1 | -12.5392 |
|  | XP\_065056045\_11954-model12.2 | -12.0994 |
|  | XP\_065056045\_11954-model12.3 | -12.0187 |
| # cluster 13 - Size: 1 |  |  |
|  | XP\_065056045\_11954-model13 | -12.0591 |
| # cluster 14 - Size: 5 |  |  |
|  | XP\_065056045\_11954-model14 | -11.7917 |
|  | XP\_065056045\_11954-model14.1 | -11.2023 |
|  | XP\_065056045\_11954-model14.2 | -9.34604 |
|  | XP\_065056045\_11954-model14.3 | -9.31788 |
|  | XP\_065056045\_11954-model14.4 | -6.67228 |
| # cluster 15 - Size: 1 |  |  |
|  | XP\_065056045\_11954-model15 | -11.5766 |
| # cluster 16 - Size: 1 |  |  |
|  | XP\_065056045\_11954-model16 | -11.3931 |
| # cluster 17 - Size: 3 |  |  |
|  | XP\_065056045\_11954-model17 | -11.3644 |
|  | XP\_065056045\_11954-model17.1 | -10.6715 |
|  | XP\_065056045\_11954-model17.2 | -8.81731 |
| # cluster 18 - Size: 2 |  |  |
|  | XP\_065056045\_11954-model18 | -11.2378 |
|  | XP\_065056045\_11954-model18.1 | -11.1698 |
| # cluster 19 - Size: 1 |  |  |
|  | XP\_065056045\_11954-model19 | -10.964 |
| # cluster 20 - Size: 1 |  |  |
|  | XP\_065056045\_11954-model20 | -10.8401 |
| # cluster 21 - Size: 1 |  |  |
|  | XP\_065056045\_11954-model21 | -8.3677 |
| # cluster 22 - Size: 1 |  |  |
|  | XP\_065056045\_11954-model22 | -7.33556 |
| # cluster 23 - Size: 2 |  |  |
|  | XP\_065056045\_11954-model23 | -7.2283 |
|  | XP\_065056045\_11954-model23.1 | -6.54794 |
| # cluster 24 - Size: 1 |  |  |
|  | XP\_065056045\_11954-model24 | -6.82558 |
| # cluster 25 - Size: 1 |  |  |
|  | XP\_065056045\_11954-model25 | -6.72127 |
| # cluster 26 - Size: 2 |  |  |
|  | XP\_065056045\_11954-model26 | -6.51347 |
|  | XP\_065056045\_11954-model26.1 | -6.37194 |
| # cluster 27 - Size: 1 |  |  |
|  | XP\_065056045\_11954-model27 | -6.36456 |
|  |  |  |
